# Early post-operative cognitive dysfunction after closed-loop versus manual target controlled-infusion of propofol and remifentanil in patients undergoing elective major non-cardiac surgery: Protocol of the randomized controlled single-blind POCD-ELA trial

**DOI:** 10.1097/MD.0000000000012558

**Published:** 2018-10-05

**Authors:** Guillaume Besch, Lucie Vettoretti, Melanie Claveau, Nathalie Boichut, Nicolas Mahr, Yannis Bouhake, Ngai Liu, Thierry Chazot, Emmanuel Samain, Sebastien Pili-Floury

**Affiliations:** a Department of Anesthesiology and Intensive Care Medicine, University Hospital of Besancon, University of Franche-Comte, Besancon; b Department of Anesthesia, Foch Hospital, Suresnes, France, and Outcomes Research Consortium, Cleveland Clinic, Cleveland, OH.

**Keywords:** anesthesia, anesthesia, general, anesthesia, intravenous, consciousness monitor, (MeSH terms) cognitive dysfunction, monitor, bispectral index

## Abstract

**Introduction:**

Post-operative cognitive dysfunction (POCD) is frequent in patients older than 60 years undergoing major non-cardiac surgery, and increases both morbidity and mortality. Anesthetic drugs may exert neurotoxic effects and contribute to the genesis of POCD. The hypothesis of the POCD-ELA trial was that closed-loop target-controlled infusion of propofol and remifentanil could reduce the occurrence of POCD by decreasing the risk of excessive depth of anesthesia and the dose of anesthetic drugs.

**Methods and analysis:**

We designed a single-center, single-blind, randomized, controlled, parallel trial and aim to include 204 patients aged >60 years undergoing elective major non-cardiac surgery. Patients will be randomized to receive closed-loop versus manual target-controlled infusion of propofol and remifentanil guided by bispectral index monitoring. Cognitive assessment will be performed the day before surgery (baseline) and within 72 hours after surgery, using a battery of validated neuropsychological tests. The primary outcome is the incidence of POCD within 72 hours after surgery. POCD is defined as a Z-score value > 1.96 for at least 2 different tests or a Z-score composite value >1.96. The calculation of the Z-score is based on data from an age-matched control population who did not undergo surgery or general anesthesia.

**Ethics and dissemination:**

This study was approved by the Ethics Committee (Comité de Protection des Personnes Est-II) and authorized by the French Health Products Agency (Agence Nationale de Sécurité des Médicaments, Saint-Denis, France). The University Hospital of Besancon is the trial sponsor and the holder of all data and publication rights. Results of the study will be submitted for publication in a peer-review international medical journal and for presentation in abstract (oral or poster) in international peer-reviewed congresses.

**Registration:**

The trial is registered with ClinicalTrials.gov (Identifier: NCT02841423, principal investigator: Prof Emmanuel Samain, date of registration: July 22, 2016). Last amendment of protocol: version 8.0 April 2018.

**Strengths and limitations of this study**

- This is the first study to investigate whether closed-loop anesthesia could decrease the rate of early post-operative cognitive dysfunction
- Single-blind randomized controlled trial.
- The definition of post-operative cognitive dysfunction is based on an alteration of cognitive performance compared to an age-matched control population who did not undergo surgery or anesthesia
- Single-center study
- No long term cognitive assessment, however early POCD is a relevant surrogate endpoint for long term cognitive impairment.

## 1 Background

Post-operative cognitive dysfunction (POCD) is a common complication that has been reported to occur in 26% of patients aged 60 years or over, up to 7 days after major non-cardiac surgery.^[1]^ POCD is usually defined as a significant alteration of cognitive function (especially memory and executive functions) during the postoperative period, compared to age- and disease-matched controls.^[1–3]^ Early POCD has been associated with increased mortality,^[4,5]^ a higher rate of dementia and premature departure from the workforce.^[5]^ Several factors have been reported to be related to POCD, but the underlying pathogenic mechanisms are complex and not fully understood.^[6]^

In particular, animal experiments have suggested that general anesthetics may exert neurotoxic effects that could lead to POCD.^[7–9]^ Since no specific treatment for POCD has been identified, prevention remains the only therapeutic measure available. In this context, anesthesia appears as a potentially modifiable contributor to POCD in the perioperative period.^[10]^ Chan et al reported that anesthesia guided by bispectral index (BIS) monitoring could decrease POCD by reducing the dose of anesthetic drugs.^[11]^ Recently, lighter anesthesia targeting higher BIS values was associated with a lower rate of POCD after total knee replacement.^[12]^ However, these data remain controversial.^[13,14]^ The discrepancies observed among the different studies could be partially explained by the difficulty in accurately maintaining the BIS value within the target range of 40 to 60 with manual titration of the anesthetic drugs. Closed-loop target-controlled infusion of propofol and remifentanil has been shown to improve the time spent within the BIS target range and to decrease both the number and the length of episodes of excessive depth of anesthesia (BIS value < 40).^[15–17]^

The hypothesis of the POCD-ELA trial was that closed-loop target-controlled infusion of propofol and remifentanil could lower the occurrence of POCD by decreasing the risk of excessive depth of anesthesia.^[18]^

## 2 Objectives

The POCD-ELA study was designed to assess whether closed-loop compared to manual target-controlled infusion of propofol and remifentanil guided by BIS monitoring could decrease the incidence of postoperative cognitive dysfunction in patients undergoing major non-cardiac surgery.

## 3 Methods and design

This manuscript is written in accordance with the SPIRIT guidelines for the reporting of interventional trial protocols.^[19]^

### 3.1 Trial design

The POCD-ELA trial is a prospective randomized, controlled, single-blind, single center study.

POCD-ELA is a sub-study of the ELA (ClinicalTrials.gov Identifier: NCT01198639) study, which aimed to assess whether closed-loop propofol-remifentanil administration compared to manual target-controlled infusion could improve 1-year mortality after non-cardiac surgery.

### 3.2 Eligibility

Adult patients scheduled for major non-cardiac surgery expected to last more than 1 hour and performed under general anesthesia are eligible for inclusion. Inclusion and exclusion criteria for the present study are detailed below. Patients undergoing emergency surgery are excluded due to the impossibility of implementing organizational aspects of the protocol in emergency conditions (see below). Patients undergoing cardiac surgery are also excluded since POCD has specific underlying mechanisms in this population.

### 3.3 Inclusion criteria

- Signed informed consent
- Age 50 to 85 years (inclusive)
- American Society of Anesthesiologists’ (ASA) physical status I to III
- Ability to speak, write and understand French language
- Scheduled non-cardiac surgery lasting more than 1 hour
- Surgically sterilized or menopausal women for at least 24 months; or, among non-menopausal women, use of an effective method of contraception.
- Patient affiliated to French Social Security or equivalent.

### 3.4 Exclusion criteria

- ASA physical status IV or higher
- Patients unable to speak, write or understand French
- Presence of a pacemaker
- Scheduled brain or cephalic surgery
- Diagnosed dementia (Alzheimer's disease, Lewy body dementia), cerebral or psychiatric pathology (tumor, stroke with sequelae, Parkinson's disease, severe depression or psychosis), as well as those receiving antipsychotic treatment (antidepressants, neuroleptics, long-term benzodiazepines, antiparkinsonian drugs)
- General anesthesia within 1 year before inclusion, except for diagnostic procedures
- Contraindication to propofol or remifentanil administration
- Legal incapacity or limited legal capacity
- Subjects without health insurance
- Pregnant or breast-feeding women
- Subjects within the exclusion period of another study.

### 3.5 Study outline

Eligible patients are screened during the anesthesia consultation and receive all information relating to the study by phone before hospital admission. Written informed consent is obtained the day before surgery by investigators after oral explanation of the study before inclusion. After written informed consent is obtained, patients are randomized.

After randomization, a first cognitive assessment is performed by a neuropsychologist blinded to the treatment allocation, using a battery of validated neuropsychological tests (see below).

Patient characteristics, past medical history including ASA physical status, and preoperative blood glucose, sodium, potassium and creatinine levels are collected. The prescription of oral premedication is at the discretion of the anesthesiologist in charge of the patient; any treatment prescribed is also recorded.

On arrival in the operating room, standard monitoring (General Electric Healthcare) is put in place. A dedicated indwelling venous catheter is inserted in a forearm vein and connected to an infusion pump via a 3-way Smartsite (Alaris Medical Systems, San Diego, CA), with a priming volume of 0.3 ml. The BIS electrode (Zipprep, Covidien, Mansfield, MA) is positioned on the patient's forehead and connected to either an A-2000 XP (version 3.11) BIS monitor (Covidien) or a BIS Module (version 4.0 XP, GE-Healthcare, Helsinki, Finland). All patients receive intravenous anesthesia with a medical device enabling calculation of effect-site concentrations of anesthetics. A standard computer is used to provide a user interface and to control communication with the BIS monitor and with both propofol and remifentanil infusion pumps (Alaris) via an RS232 serial port (Infusion Toolbox 95w version 4.11 software (Department of Computer Science, Faculty of Medicine, Université Libre de Bruxelles, Brussels)). The protocol for general anesthesia depends on the treatment allocated by randomization, that is, closed-loop (intervention group) or manual (conventional group) target-controlled infusion of propofol and remifentanil guided by BIS monitoring (see below). With the exception of propofol and remifentanil delivery, the anesthetic protocol is at the discretion of the anesthesiologist in charge of the patient.

Surgical and anesthetic details are recorded, including: type and duration of surgery; intraoperative blood loss and urine output, blood transfusion, total volume of fluids infused; type and total dose of drugs administered; and all blood pressure, heart rate and pulse oximetry values measured from induction of anesthesia to discharge from the operating room. All data concerning BIS monitoring and propofol and remifentanil delivery are extracted from the computer connected to the BIS monitor and to the propofol and remifentanil infusion pumps. Post-operative pain management and assessment is recorded.

A second cognitive assessment is performed by a neuropsychologist blinded to the treatment allocation within 72 hours after the end of surgery, using the same battery of validated neuropsychological tests as during the first evaluation (see below). If the patient refuses to participate in the second cognitive assessment, the consent is considered to be withdrawn, and the patient is excluded from the study.

### 3.6 Randomization, allocation concealment and blinding

Centralized randomization is performed using a specialised website on the day before surgery. A computer-generated permuted-block randomization list with varying block sizes (ratio 1:1, block size of 2 and 4) was implemented on the website at the beginning of the ELA-study by an independent data manager. The block size is unknown to the investigators. The investigator enters data online regarding each patient to enable inclusion, and the treatment randomly assigned to the patient is immediately provided to the investigator by the website. The allocation ratio is 2:1 (interventional: conventional groups), and the randomization is stratified by age (> or <70 years) and cancer (yes/no). The allocation ratio and the stratification are the same as in the ELA study.

Patients and outcome assessors (neuropsychologists) are blinded to the treatment allocated.

### 3.7 Study procedure and interventions

In the interventional group, anesthesia is performed using a dual closed-loop controller allowing automated propofol–remifentanil titration guided by BIS. A computer plays the role of an interface between the BIS monitor and the propofol and remifentanil infusion pumps (Alaris Medical, Hampshire, UK) via an RS232 serial port (Infusion Toolbox 95w version 4.11 software). Propofol and remifentanil are automatically titrated by the controller to maintain a BIS value as close as possible to 50 and between 40 and 60 (target range).^[15,20]^ The BIS electrode impedance is checked regularly, and the BIS sampling rate is 256 Hz with a 15 s smoothing rate. The BIS value is considered valid when the signal quality index is >50. The clinical effectiveness and safety of the closed-loop controller have previously been published elsewhere.^[15]^

In the conventional group, propofol and remifentanil are manually titrated by the anesthesiologist and the nurse anesthesiologist in charge of the patient to maintain a BIS value within the target range. In the conventional group, the BIS monitor and the propofol and remifentanil infusion pumps are also connected to the computer to allow data collection regarding the management of anesthesia with the same precision as in the interventional group.

Intraoperative ketamine is forbidden in the 2 groups to avoid biased estimation of the depth of anesthesia by BIS monitoring.

### 3.8 Outcome measures

The primary outcome is the incidence of POCD within 72 hours after major non-cardiac surgery under general anesthesia. A cognitive assessment is performed the day before surgery (baseline) and within 72 hours after surgery using a battery of validated neuropsychological tests (see below) during a face-to-face interview with a trained neuropsychologist. To avoid potential underestimation of the cognitive status, the evaluation is conducted in a quiet environment, after checking for the absence of uncontrolled pain, hypoglycemia, or dysnatremia. The cognitive assessment includes 7 neuropsychological tests that explore 6 different cognitive domains:

1. the global cognitive functioning using the Mini-Mental State Examination (MMSE)^[21]^ and the Mattis Dementia Rating Scale,^[22]^
2. attention, using the Trail Making Test, part A (TMT-A),
3. executive function, using the Trail Making Test, part B (TMT-B) and Stroop tests,
4. verbal fluency, using the Isaacs Set Test (IST),^[23]^
5. learning and episodic verbal memory, using the Memory Impairment Screen (MIS) test,^[24,25]^
6. a psychomotor task using the crossing-off test.^[26]^

According to the method described by Moller et al,^[1,3]^ a Z-score value will be calculated for each neuropsychological test as follows: Z-score = (X1–X2)/SD; where X1 and X2 are the score values obtained at baseline and at the 2^nd^ assessment respectively; and Standard deviation (SD) is the SD of the test performed in an age-matched control population who did not undergo surgery or general anesthesia. The SD observed in the control population is known for all tests used. A composite Z-score value will be calculated as the mean of the Z-scores of all tests.

POCD is defined as a Z-score value >1.96 for at least 2 different tests or a Z-score composite value >1.96.

Secondary outcomes are the differences in the score obtained on each test between baseline and post-surgery assessment.

### 3.9 Laboratory measurements

Preoperative creatinine, blood glucose, natremia, and kalemia measurements are at the discretion of the anesthesiologist in charge of the patient and will be recorded when available.

Blood creatinine, blood glucose, natremia, and kalemia are measured on the day of the second assessment of the cognitive function to ensure absence of confusion, which may lead to an overestimation of the primary outcome.

### 3.10 Safety

All serious adverse events are collected and reviewed by the principal investigator and reported to the trial sponsor (CHU Besancon, Besancon, France) and to the Pharmacovigilance Department of Our Institution. Study insurance has been contracted for all participating subjects by the trial sponsor (CHU Besancon, Besancon, France).

Since no serious adverse event related to patient participation is expected in this study, no independent data safety monitoring committee has been established.

### 3.11 Sample size calculation

The difference in primary outcome between groups will be compared using Pearson's Chi-squared test, based on an intention-to-treat analysis. Based on the data from the ISPOCD1 study,^[1]^ the expected rate of POCD in the conventional group is 26%. We assume that the expected rate of POCD in the interventional group (closed-loop infusion of propofol and remifentanil) is 10%. Considering a loss to follow-up rate of 5%, a total sample size of 204 patients was calculated (68 patients in the conventional arm, 136 patients in the interventional arm), on a final analysis using the Pearson's Chi-squared test (2-sided, power 80%, alpha 0.05) using the SAS software, version 9.4 (SAS Institute, Inc.).

### 3.12 Statistical analysis

The difference in primary outcome will be compared using Pearson Chi-squared test, based on per-protocol analysis. The per-protocol population is defined as any patient who undergoes the 2 assessments of the cognitive function, that is, at baseline and within 72 hours after surgery. No interim or subgroup analysis is planned.

The normality of the distribution of continuous variables will be tested by using the Shapiro–Wilk test. Intergroup comparisons will be conducted by using the Mann–Whitney *U* or Student *t* tests for quantitative variables, depending on the distribution of data, and using Fisher exact test or the Chi-squared test for qualitative variables.

A logistic regression will be performed to model the risk of POCD including variables with a *P* value of <.20 in the univariate analysis and the treatment allocated.

### 3.13 Monitoring

The Department of Research and Clinical Investigation of our institution will monitor all written informed consent, inclusion and exclusion criteria, and check all serious adverse events.

## 4 Ethics and dissemination

### 4.1 Ethics approval and registration

This study was approved by the French Ethics Committee (Comité de Protection des Personnes Est-II, CHU de Besancon, Chairperson Pr Eric TOUSSIROT, N°12/654 on 05-nov-2012) and authorized by the French Health Authority (Agence Nationale de Sécurité du Médicament—N°2012-A00557-36 on 25-jan-2013). The POCD-ELA study is registered with ClinicalTrials.gov (Identifier: NCT02841423, principal investigator: Prof Emmanuel Samain, date of registration: July 22, 2016). This study is carried out in accordance with GCP-ICH-6 and conducted in a single university-affiliated hospital (CHU de Besancon, Besancon, France). Eligible patients were screened during the anesthesia consultation and received all information relating to the study during a phone call with investigators before hospital admission. Written informed consent was obtained by investigators before inclusion the day before the surgery.

### 4.2 Planning and dissemination

The study started inclusions on February 4, 2013. The initial planned duration of the trial was 2 years. Protocol amendments for study prolongation have been approved by the French Ethics Committee and communicated to all investigators and trial registries. The university hospital of Besancon (CHU Besancon, Besancon, France) is the trial sponsor and the holder of all data and publication rights. The results of the study will be submitted for publication in a peer-review international medical journal and presented at abstract form in national and international conferences.

## Author contributions

Guillaume Besch: study design and writing of the manuscript; future analysis and interpretation of study data.

Lucie Vettoretti: writing of the manuscript; future acquisition and analysis of study data.

Melanie Claveau: conception and study design; critically reviewing the manuscript; future analysis and interpretation of study data.

Nathalie Boichut: critically reviewing the manuscript; future acquisition and interpretation of study data.

Nicolas Mahr: critically reviewing the manuscript; future acquisition and interpretation of study data.

Yannis Bouhake: critically reviewing the manuscript; future acquisition and interpretation of study data.

Ngai Liu: conception and study design; critically reviewing the manuscript; future analysis and interpretation of study data.

Thierry Chazot: conception and study design; critically reviewing the manuscript; future analysis and interpretation of study data.

Emmanuel Samain: conception and study design; critically reviewing the manuscript; future analysis and interpretation of study data.

Sebastien Pili-Floury: conception and study design; critically reviewing the manuscript; future analysis and interpretation of study data.

All authors approved the final version of the manuscript to be published and are accountable for all aspects of the work.

**Conceptualization:** Mélanie Claveau, Nathalie Boichut, Ngai Liu, Thierry Chazot, Emmanuel Samain, Sébastien Pili-Floury.

**Data curation:** Emmanuel Samain, Sébastien Pili-Floury.

**Formal analysis:** Guillaume Besch, Lucie Vettoretti, Nathalie Boichut, Nicolas Mahr, Yannis Bouhake, Emmanuel Samain, Sébastien Pili-Floury.

**Funding acquisition:** Mélanie Claveau, Nathalie Boichut, Emmanuel Samain, Sébastien Pili-Floury.

**Investigation:** Mélanie Claveau, Nathalie Boichut, Nicolas Mahr, Yannis Bouhake, Emmanuel Samain, Sébastien Pili-Floury.

**Methodology:** Guillaume Besch, Nathalie Boichut, Ngai Liu, Thierry Chazot, Emmanuel Samain, Sébastien Pili-Floury.

**Project administration:** Guillaume Besch, Lucie Vettoretti, Mélanie Claveau.

**Resources:** Lucie Vettoretti, Ngai Liu, Thierry Chazot.

**Software:** Ngai Liu, Thierry Chazot.

**Supervision:** Guillaume Besch, Nathalie Boichut, Emmanuel Samain, Sébastien Pili-Floury.

**Validation:** Guillaume Besch, Mélanie Claveau, Nicolas Mahr, Yannis Bouhake, Ngai Liu, Thierry Chazot, Emmanuel Samain, Sébastien Pili-Floury.

**Visualization:** Guillaume Besch, Lucie Vettoretti, Nicolas Mahr, Yannis Bouhake, Ngai Liu, Thierry Chazot, Emmanuel Samain, Sébastien Pili-Floury.

**Writing – original draft:** Guillaume Besch, Lucie Vettoretti, Emmanuel Samain, Sébastien Pili-Floury.

**Writing – review & editing:** Guillaume Besch, Mélanie Claveau, Nathalie Boichut, Nicolas Mahr, Yannis Bouhake, Ngai Liu, Thierry Chazot, Emmanuel Samain, Sébastien Pili-Floury.
